# Phantom-based quantification of the spectral accuracy of lower extremity vascular imaging using a second-generation dual-layer spectral CT at 100 kVp and 120kVp

**DOI:** 10.1186/s12880-025-02047-8

**Published:** 2025-12-04

**Authors:** Xinyu Gao, Chao Zhang, Yun Wang, Xiaomei Lu, Shenghui Yu, Wenjie Zheng, Zehong Lin, Yining Wang, Huadan Xue, Daming Zhang, Zhengyu Jin

**Affiliations:** 1 Department of Radiology, Peking Union Medical College Hospital, Peking Union Medical College and Chinese Academy of Medical Sciences, No.1 Shuaifuyuan, Wangfujing Street, Dongcheng District, Beijing, 100730 China; 2 Peking Union Medical College and Chinese Academy of Medical Sciences, No. 9 Dongdan Santiao, Dongcheng District, Beijing, 100730 China; 3 CT Clinical Science CT, Philips Healthcare, Beijing, 100600 China; 4 CT Clinical Technical Support, Philips Healthcare, Beijing, 100600 China; 5 Department of Rheumatology and Immunology, Peking Union Medical College Hospital, Peking Union Medical College and Chinese Academy of Medical Sciences, No. 1 Shuaifuyuan, Wangfujing Street, Dongcheng District, Beijing, 100730 China; 6 College of Civil and Architectural Engineering, Harbin University, Harbin, China

**Keywords:** Phantom imaging, Lower limb vessel, X-ray computed tomography, Radiation dose

## Abstract

**Background:**

This study quantitatively evaluated the effects of tube voltage, CTDI_vol_, energy level, and iodine concentration on the performance of lower-extremity vascular imaging using a second-generation dual-layer spectral CT (DSCT). We further assessed the potential for reducing radiation dose and contrast agent use without compromising image quality.

**Methods:**

A spectral CT phantom with tissue-equivalent inserts and varying iodine concentrations (4, 8, 12, and 16 mgI/mL) was scanned at different tube voltages (100/120 kVp), CTDI_vol_ levels (2.5, 5, 10 mGy), and virtual monoenergetic levels (40, 70, 100 keV), alongside conventional imaging. Vessel diameters of 2.5, 4, and 5 mm were evaluated. Objective image quality was assessed via CT attenuation, SD, SNR, and CNR. Two radiologists used a 5-point Likert scale to assess the subjective image quality.

**Results:**

No significant differences were observed in subjective or objective scores between tube voltages or across CTDI_vol_ levels (*p* > 0.05). Low-energy 40 keV images yielded comparable quality to 70 keV and conventional images. At 40 keV, ultra-low iodine concentrations (4 mgI/mL) achieved image quality similar to higher concentrations (12/16 mgI/mL) in conventional scans. The combination of 100 kVp, 2.5 mGy, and 40 keV provided superior image quality relative to conventional CT.

**Conclusions:**

Second-generation DSCT at 100 kVp provides high-accuracy lower-limb vascular imaging, even at low radiation doses and contrast concentrations, supporting its potential for clinical dose-reduction strategies.

**Supplementary Information:**

The online version contains supplementary material available at 10.1186/s12880-025-02047-8.

## Background

Peripheral artery disease (PAD) is a major cause of disability and mortality worldwide [1], with increasing incidence linked to aging and better survival rates from coronary artery disease and stroke [2]. PAD is characterized by atherosclerotic stenosis or occlusion of peripheral arteries, usually in the lower limbs [1]. Computed tomography angiography (CTA) is the preferred diagnostic method for PAD due to its availability, non-invasive nature, rapid image acquisition, effectiveness, and high diagnostic accuracy [3]. Despite these advantages, many patients are deterred by computed tomography (CT) radiation doses and the adverse effects of contrast agents. Due to the large scanning range, bilateral lower-limb CTA scans require higher radiation and contrast agent dose compared other body organs [4]. PAD is associated with impaired renal function, and many patients requiring CTA cannot tolerate the high contrast medium (CM) doses typically needed for lower-extremity imaging. Reducing CM doses is crucial to lowering the risk of acute kidney injury. Lowering the tube voltage can effectively reduce the radiation dose, as tube voltage is proportional to the 2.5–3.1 exponent of the radiation dose [5].

Among commercially available dual-energy CT (DECT) systems, dual-layer spectral CT uniquely creates spectral separation at the detector level. This method provides key advantages: simultaneous generation of spectral and conventional images, spatial and temporal alignment of both spectra, reduced beam-hardening artifacts [6], and better spectral denoising [7]. A recent study reported higher accuracy of second-generation spectral CT than first-generation spectral CT at identical radiation doses and a collimation width of 4 cm [8]. Existing literature [8, 9] has demonstrated that second-generation dual-layer spectral CT significantly reduces scattered radiation compared to their first-generation dual-layer spectral CT counterparts. While spectral information’s clinical benefits are well-established [10], its application in lower-extremity vessels remains underexplored.

First-generation DECT requires spectral imaging at 120 or 140 kVp [11, 12], and the second-generation DECT can achieve spectral imaging at 100 kVp, improving confidence in the clinical application of lower extremity CTA with low tube voltage. Virtual monoenergetic images (VMI), especially below 60 keV, improve iodine attenuation and vascular imaging contrast, potentially salvaging suboptimal scans [13]. While numerous studies have evaluated lower extremity artery imaging with first-generation DECT [14], few studies have focused on second-generation DECT. This phantom study aimed to quantitatively investigate the impact of different tube voltages, Computed tomography dose index volume (CTDI_vol_), energy levels, and contrast agent concentrations on spectral imaging performance for lower-limb vessels imaging using DECT. We also explored whether radiation dose and contrast agent concentration could be reduced without compromising image quality.

## Methods

### Phantom configuration

**Fig. 1:**
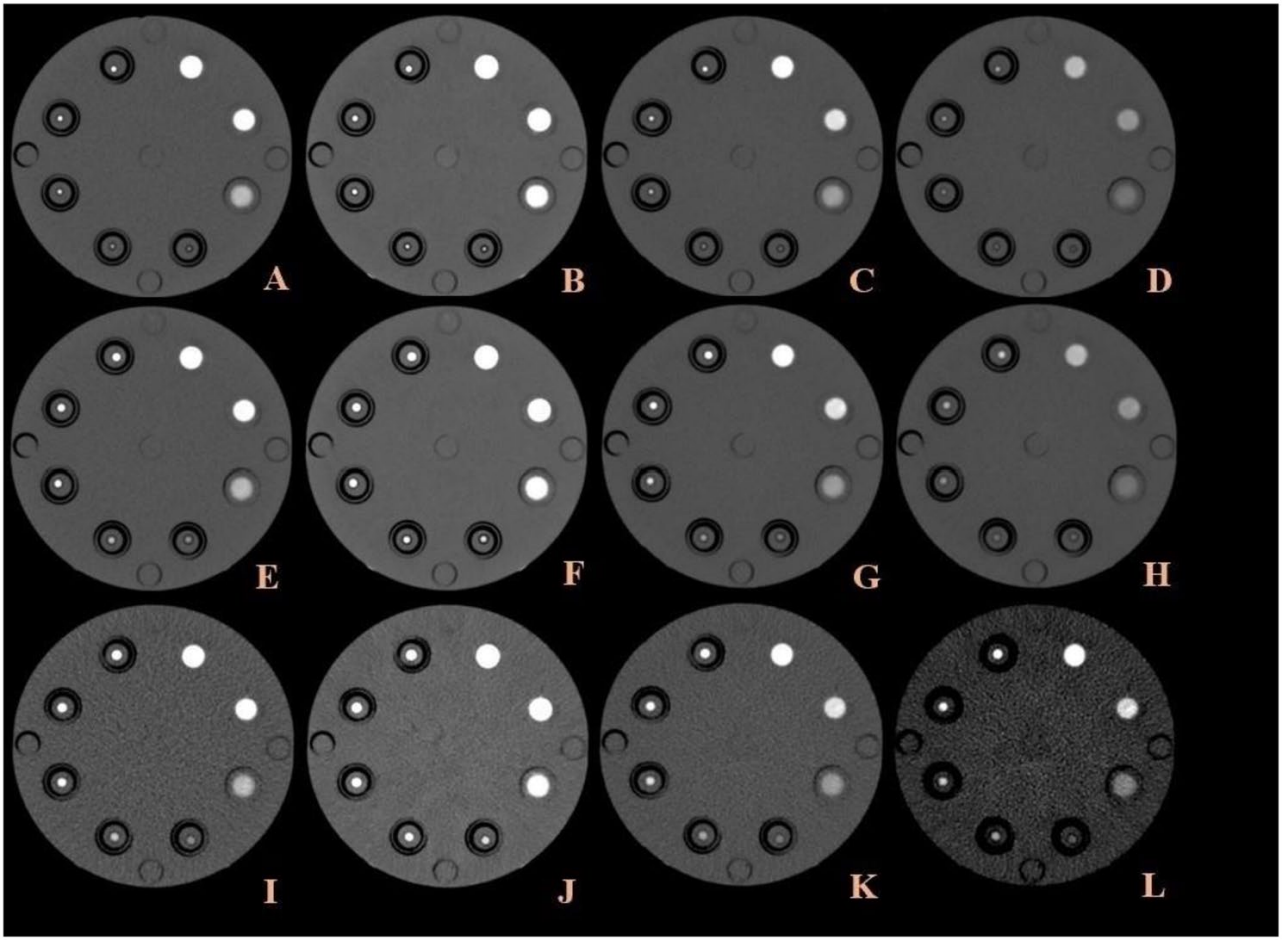
Cross-sectional pictures of phantoms with different conditions. **A**-**D**: The tube voltage was 100kVp and the insert diameter was 2.5 mm. Conventional CT scanning(**A**) and virtual monochromatic images at 40 keV(**B**), 70 keV(**C**), 100 keV(**D**) are shown. **E**-**H**: The tube voltage was 100kVp and the insert diameter was 4 mm. Conventional CT scanning(**E**) and virtual monochromatic images at 40 keV(**F**), 70 keV(**G**), 100 keV(**H**) are shown. **I**-**L**: The tube voltage was 100kVp and the insert diameter was 5 mm. Conventional CT scanning (**I**) and virtual monochromatic images at 40 keV(**J**), 70 keV(**K**), 100 keV(**L**) are shown

We used a spectral CT phantom (PH-75; Kyoto Kagaku), consisting of a solid water disk with a diameter of 16 cm and a thickness of 18 cm. The phantom contained a concentric ring with eight holes. All inserts measured 2.0 cm in diameter and 8.0 cm in length (Fig. 1). Phantom models with three different tube diameters and five different iodine concentrations were placed in sample tubes attached to the phantom model. The phantom diameters were 2.5, 4, and 5 mm, and the iodine concentrations were 2, 4, 8, 12 and 16mgI/mL, respectively. The iodine concentrations of 2 mgI/mL were excluded in subsequent analyses due to poor image quality.

### Data acquisition

All acquisitions were performed using a commercial dual-layer spectral CT scanner (Spectral CT 7500, Philips Healthcare) at three radiation dose levels: CTDI_vol_ of 2.5, 5, and 10mGy each at tube voltages of 100 and 120 kVp. For each tube voltage setting, the tube current-time product (mAs) was adjusted to achieve the targeted CTDI_vol_. Other scanning and reconstruction parameters were fixed, and a summary of all acquisitions is presented in Table 1. For each scenario (i.e., a combination of iodine concentration and phantom size), images were obtained at three different dose levels (CTDI_vol_: 2.5, 5, and 10mGy). In total, 18 datasets (two tube voltages × three dose levels ×three repetitions [15, 16]) were collected for each phantom configuration. No institutional review board approval was required as this was a phantom study.

**Table 1:** Dual-layer spectral CT acquisition and reconstruction parameters

Measures	Values
Pipe diameter (mm)	2.5, 4, 5
Tube voltage (kVp)	100, 120
Collimation width (mm)	64*0.625
Dose level (CTDI_vol_)(mGy)	2.5, 5, 10
Tube current (mAs)	Auto mAs
Pitch	0.81
Slew time (s)	0.75
Slice thickness (mm)	3
Reconstruction matrix	512*512
Iodine concentrations of phantom (mgI/mL)	4, 8, 12, 16
Tube diameter of phantom (mm)	2.5, 4, 5

### Image quality assessment

All reconstructed spectral data were imported into a workstation (Intelli Space Portal, Philips Healthcare) for measurements. The spectral data from each group were opened using the Spectral CT View tool, and the image window width and level were fixed at the preset CTA. During measurement, the region of interest (ROI) was placed in the center of each simulated blood vessel, covering 80% of the vessel area. A Spectral Plot was used to obtain and record the CT_blood vessel_ and standard deviation _blood vessel_ (SD_blood vessel_ ) for each ROI at different energy levels (40, 70, 100 keV). The CT_blood vessel_ and SD_blood vessel_ for each ROI in conventional CT were also recorded. A uniform tissue sample, without filling in each tissue rod, was selected to repeat the measurements, fix the ROI size, and measure its CT value and standard deviation, representing the CT_background_ valus and SD_background_. Simultaneously, the signal-to-noise ratio (SNR) and contrast-to-noise ratio (CNR) for each ROI were calculated, using the formulas below.

$$\:SNR={CT}_{\text{b}\text{l}\text{o}\text{o}\text{d}\:\text{v}\text{e}\text{s}\text{s}\text{e}\text{l}}/{SD}_{blood\:vessel}$$

$$\begin{aligned}CNR=&\:\left({CT}_{\text{b}\text{l}\text{o}\text{o}\text{d}\:\text{v}\text{e}\text{s}\text{s}\text{e}\text{l}}-{CT}_{background}\right)\cr &/{SD}_{background}\end{aligned}$$

Two experienced radiologists (each with ≥ 3 years of diagnostic experience) independently assessed image quality using a 5-point Likert scale (1–2: poor contrast, very blurred lumen edge and high noise, not suitable for diagnose; 3: suboptimal contrast, blurred lumen edge and high noise, sufficient to exclude obstructive disease; 4: good contrast, sharp lumen edge, and low noise, suitable for evaluating the degree of stenosis and identifying mild atherosclerosis; and 5: excellent contrast, very sharp lumen edge, and very low noise; allowing for simultaneous evaluation of both obstructive disease and mild atherosclerosis) [17] under double-blind conditions. Radiologists could adjust the window width and window level for qualitative image assessment. The radiologists were blinded to the reconstruction protocol and scanning conditions. In case of disagreement, the two doctors reached a consensus through discussion.

### Statistical analysis

Statistical analyses were performed using SPSS Statistics software (version 21.0, IBM). Continuous variables are expressed as mean ± SD. We employed one-way analysis of variance (ANOVA) and paired t-test adjusted with the Bonferroni correction, or independent t tests to compare the objective evaluation indices (mean CT value, SD, SNR value, and CNR value). The Kappa test was used to assess inter-reader agreement in the subjective evaluation of image quality. For subjective evaluation indicators (contrast, sharpness, subjective noise, and acceptability) [17], the Kruskal-Wallis H test was applied, and the Mann-Whitney U test with Bonferroni correction was applied for multiple comparisons. A value of *P* < 0.05 was considered statistically significant.

## Results

### Comparison of image quality at different tube voltages

**Fig. 2:**
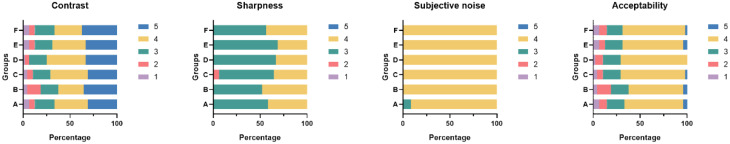
Subjective image quality score of different tube voltages at three different CTDI_vol_. Group A: 100kVp and 2.5 mGy, Group B: 120kVp and 2.5 mGy, Group C: 100kVp and 5 mGy, Group D: 120kVp and 5 mGy, Group E: 100kVp and 10 mGy, Group F: 120kVp and 10 mGy

The subjective ratings by the two radiologists were consistent across groups. The Kappa values for image contrast, sharpness, subjective noise, and acceptability were 0.80, 0.86, 0.83, and 0.85, respectively. First, we used the Mann-Whitney U test to compare the subjective evaluation scores between the 100 kVp and 120 kVp groups. There were no significant differences in contrast, sharpness, subjective noise, or acceptability between the two groups (U = 10162.500, 9666.000, 101152.000, and 10110.500, *P* = 0.761, 0.243, 0.082, and 0.666, respectively). Second, we compared the subjective evaluation scores at 100kVp and 120 kVp across three different computed tomography dose indices (CTDI_vol_: 2.5 mGy, 5 mGy, 10 mGy). No significant differences were found in contrast (U = 1140.500, 1092.500, and 1132.000, respectively, *P* = 0.930, 0.644, and 0.878, respectively), sharpness (U = 1080.000, 1128.000, and 1008.000, respectively. *P* = 0.540, 0.835, and 0.208, respectively), subjective noise (U = 1080.000, 1152.000, and 1152.000, respectively. *P* = 0.080, 1.000, 1.000, respectively) or acceptability (U = 1103.500, 1137.500, and 1129.500, respectively. *P* = 0.686, 0.895, and 0.845, respectively) between the two tube voltage groups(Table S1, Fig. 2).

**Fig. 3:**
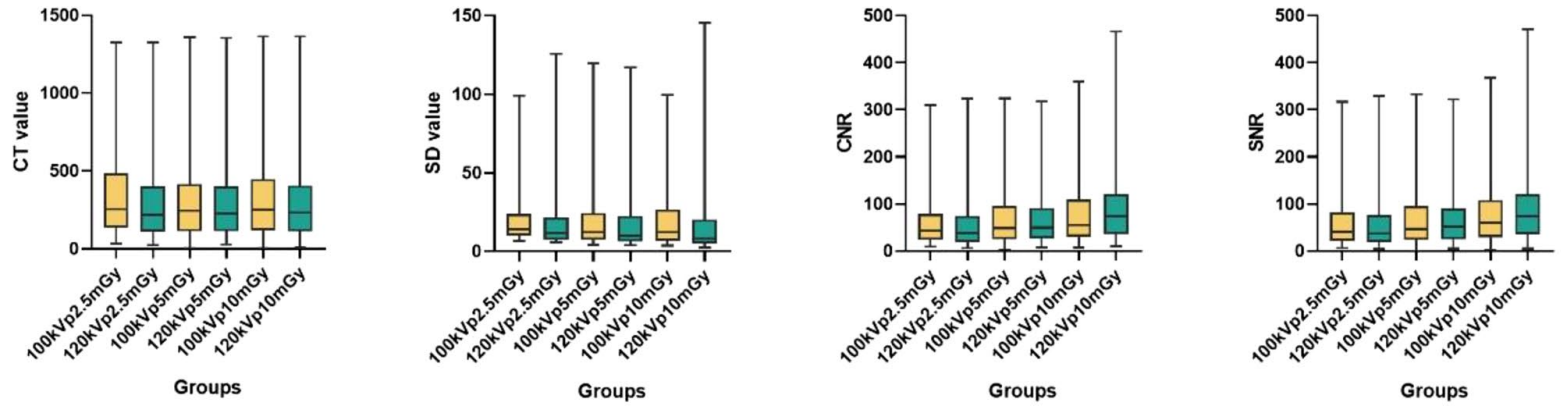
Comparison of objective evaluation indexes of different tube voltages at three different CTDI_vol_. Box plots show CT value, SD, CNR and SNR obtained under different tube voltages (100kVp and 120kVp) and CTDI_vol_ (2.5mGy, 5mGy, and 10mGy) levels. The box represents the interquartile range (IQR) from Q1 to Q3, with the horizontal line indicating the median

We also conducted a comparison of objective evaluation indices for image quality. There were no significant differences in CT, SD, CNR, and SNR values between the 100 kVp and 120 kVp groups (t = 0.419, 0.350, -0.562, and − 0.593, respectively; *P* = 0.676, 0.726, 0.575, and 0.554, respectively.). Similarly, there were no significant differences in CT (t = 0.330, 0.170, and 0.225, respectively; *P* = 0.742, 0.865, and 0.823, respectively), SD (t = 0.094, 0.220, and 0.275, respectively; *P* = 0.926, 0.826, and 0.784, respectively), CNR (t=-0.079, -1.174, and − 0.620, respectively; *P* = 0.937, 0.862, and 0.537, respectively) and SNR (t=-0.091, -0.197, and − 0.639, respectively; *P* = 0.928, 0.844, and 0.524, respectively) values between the 100 kVp and 120 kVp groups across the three CTDI_vol_ levels (2.5 mGy, 5 mGy, and 10 mGy) (Table S2, Fig. 3).

At 100kVp, there were no significant differences in CT, SD, CNR, and SNR values across the three different CTDI_vol_ levels (2.5 mGy, 5 mGy, and 10 mGy) (F = 0.003, 0.001, 2.156, and 1.995, respectively; *P* = 0.997, 0.999, 0.120, and 0.140, respectively). Similarly, at 120 kVp, no significant differences were observed in CT, SD, and SNR values across the three CTDI_vol_ levels (F = 0.005, 0.027, and 3.022, respectively; *P* = 0.995, 0.973, and 0.052, respectively). The one-way ANOVA revealed a statistically significant difference in CNR values across the three CTDI_vol_ groups (F = 3.128, *P* = 0.047). Post hoc analysis with Bonferroni correction indicated that the CNR in the 2.5 mGy group was significantly lower than that in the 10 mGy group. (Table S3, Fig. 3).

### Comparison of image quality at different KeV levels

At 100 kVp and 2.5 mGy, we compared subjective evaluation indices at different energy levels (40 keV, 70 keV, 100 keV, and conventional CT). The three monoenergetic image series were 40 keV (Group A), 70 keV (Group B), and 100 keV (Group C), while conventional CT images were defined as Group D. Subjective evaluation indicators (contrast, sharpness, subjective noise, and acceptability) from the four groups were compared using the Kruskal-Wallis H test, with post hoc pairwise Mann-Whitney U tests employing Bonferroni correction. The Kruskal-Wallis H test indicated statistically significant differences in contrast (χ^2^ = 19.395, *P* < 0.001), subjective noise (χ^2^ = 12.818, *P* = 0.005), and acceptability (χ^2^ = 17.685, *P* = 0.001) across the four groups. The Bonferroni-corrected p-values for the pairwise Mann-Whitney U tests are presented in Table 2. The significance level was set at α = 0.05.

**Table 2:** Comparison of subjective observation indices of image quality among all groups

Item	Group A 40 keV (1/2/3/4/5)^#^	Group B 70 keV (1/2/3/4/5)	Group C 100 keV (1/2/3/4/5)	Group D conventional (1/2/3/4/5)	Corrected statistical value(A vs. B)	Corrected statistical value(A vs. C)	Corrected statistical value (A vs. D)
Contrast	0/0/0/5/7°	0/0/3/5/4	3/3/3/3/0	0/0/4/4/4	U = 46.500 *P* = 0.318	U = 7.500 *P*<0.001	U = 44.000 *P* = 0.237
Sharpness	0/0/6/6/0	0/0/8/4/0	0/0/5/7/0	0/0/9/3/0	U = 60.000 *P* = 1.000	U = 66.000 *P* = 1.000	U = 54.000 *P* = 0.648
Subjective noise	0/0/0/12/0	0/0/0/12/0	0/0/0/12/0	0/0/4/8/0	U = 72.000 *P* = 1.000	U = 72.000 *P* = 1.000	U = 48.000 *P* = 0.096
Acceptability	0/0/0/11/1	0/1/2/8/1	3/3/3/3/0	0/0/4/8/0	U = 55.500 *P* = 0.537	U = 16.5000 *P*<0.001	U = 44.000 *P* = 0.066

^#^ (1/2/3/4/5) represents the scores of each subjective evaluation index. ° For example, 0/0/0/5/7 represents the number of each subjective score

**Fig. 4:**
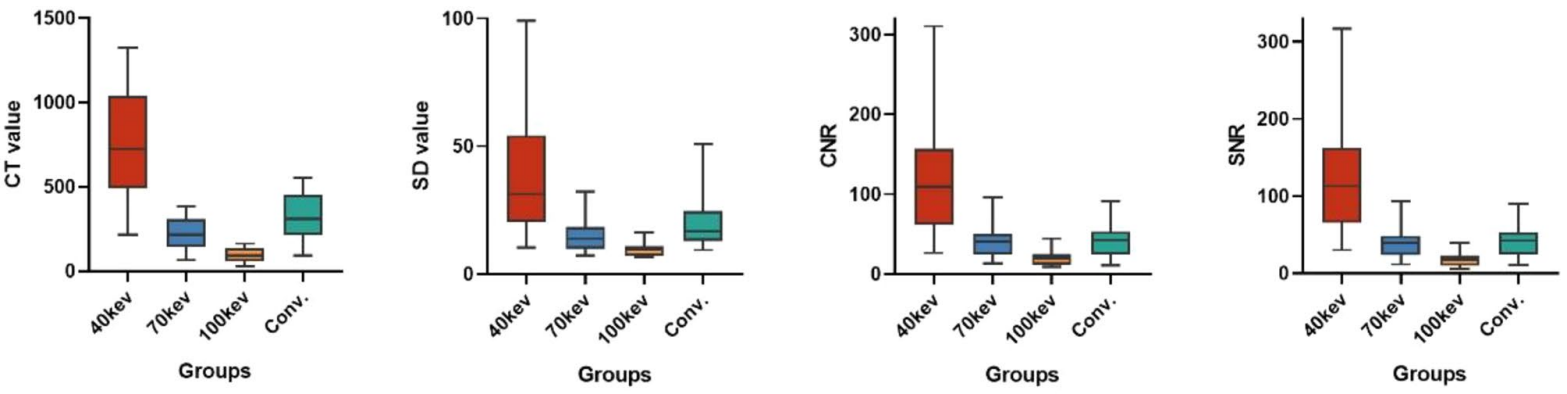
Comparison of objective indexes of different energy levels at 100kVp 2.5mGy. Box plots show CT value, SD, CNR and SNR under different energy level (40 keV, 70 keV, and 100 keV), as well as those obtained with conventional CT. The box represents the interquartile range (IQR) from Q1 to Q3, with the horizontal line indicating the median

We then compared objective evaluation indices (CT, SD, CNR, and SNR) at different energy levels (40 keV, 70 keV, 100 keV, and conventional CT) at 100 kVp and 2.5 mGy. The one-way ANOVA demonstrated a statistically significant difference in the CT values (F = 26.369, *P*<0.001), SD (F = 8.349, *P*<0.001), CNR(F = 11.885, *P*<0.001), and SNR(F = 13.148, *P*<0.001) values among the four energy level groups. Group A (40 keV) had higher CT values and a higher SNR than the other groups (Table 3; Fig. 4).

**Table 3:** Objective evaluation indexes of different energy levels at 100kVp 2.5mGy

Value	A (40 keV)	B (70 keV)	C (100 keV)	D (Conventional)	*P* value
CT	626.2 ± 181.4*^#^°	182.0 ± 53.0^&^	75.9 ± 22.4^&^°	269.5 ± 78.3^&#^	<0.001
SD	64.1 ± 13.7*^#^°	20.9 ± 4.2^&^	10.8 ± 1.9^&^	33.6 ± 6.5^&^	<0.001
CNR	142.8 ± 62.3*^#^°	47.8 ± 17.6^&^	23.1 ± 7.5^&^	47.7 ± 16.8^&^	<0.001
SNR	147.9 ± 63.0*^#^°	45.9 ± 17.5^&^	19.3 ± 7.3^&^	47.3 ± 16.8^&^	<0.001

Pairwise comparisons with Bonferroni correction showed significant differences from 40 keV (^&^), 70 keV (*), 100keV (^#^), conventional CT (°) (*P*<0.05). Abbreviation: CT, computed tomography value. SD, standard deviation. SNR, signal-to-noise ratio. CNR, contrast-to-noise ratio

### Comparison of image quality at different iodine concentrations

**Fig. 5:**
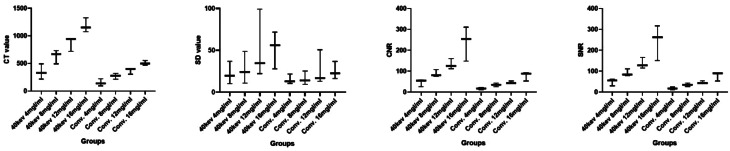
Comparison of objective evaluation indexes of different Iodine concentrations at 40 keV and conventional energy levels. Box plots show CT value, SD, CNR and SNR at different energy levels and iodine concentrations. The box represents the interquartile range (IQR) from Q1 to Q3, with the horizontal line indicating the median

We compared the image quality of different iodine concentrations at both conventional and 40 keV levels under low tube voltage (100 kVp) and low CTDI_vol_ (2.5 mGy). Table 4 presents the CT value, SD, CNR, and SNR values for all contrast agent concentrations (4, 8, 12, 16 mgI/mL) under both 40 keV and conventional CT settings. We compared the subjective and objective scores among Group 1 (40 keV, 4 mgI/mL), Group 2 (conventional CT, 12 mgI/mL), and Group 3 (conventional CT, 16 mgI/mL) using ANOVA and Kruskal-Wallis H tests, respectively. The results showed no statistically significant differences in CT (F = 2.703, *P* = 0.146), SD (F = 0.071, *P* = 0.932), CNR (F = 3.904, *P* = 0.082), and SNR (F = 3.463, *P* = 0.100) values among Groups 1 (40 keV, 4mgI/mL), 2 (conventional, 12mgI/mL), and 3 (conventional, 16mgI/mL) (Fig. 5). Similarly, there was no significant difference in contrast (χ^2^ = 5.600, *P* = 0.061), sharpness (χ^2^ = 4.571, *P* = 0.102), subjective noise (χ^2^ = 2.000, *P* = 0.368), and acceptability (χ^2^ = 0.000, *P* = 1.000) among the three groups.

**Table 4:** Objective evaluation indexes of different iodine concentrations at different energy levels

Value	4mgI/mL		8mgI/mL		12 mgI/mL		16 mgI/mL	
	40 keV	Conventional	40 keV	Conventional	40 keV	Conventional	40 keV	Conventional
CT	345.52 ± 138.07	150.48 ± 63.52	631.77 ± 123.97	270.70 ± 53.43	869.02 ± 128.50	370.62 ± 58.57	1182.81 ± 127.84	504.83 ± 43.97
SD	22.26 ± 13.41	15.04 ± 5.96	27.95 ± 19.31	16.31 ± 8.18	51.97 ± 41.32	26.97 ± 20.73	51.81 ± 22.28	25.23 ± 10.39
CNR	45.91 ± 16.95	17.60 ± 6.43	86.94 ± 17.11	34.98 ± 8.46	132.74 ± 24.57	45.91 ± 6.06	237.69 ± 81.99	77.55 ± 21.10
SNR	49.33 ± 16.77	17.59 ± 6.74	90.91 ± 17.21	34.90 ± 8.09	136.49 ± 26.53	46.27 ± 6.35	243.38 ± 84.18	77.51 ± 21.19

Values in the table are presented as mean ± standard deviation. Abbreviation: CT, computed tomography value. SD, standard deviation. SNR, signal-to-noise ratio. CNR, contrast-to-noise ratio

### Comparison of image quality at different tube diameters

We compared the image quality of different tube diameters at conventional and 40 keV levels under low tube voltage (100 kVp) and low CTDI_vol_ (2.5 mGy). The results showed that at the conventional CT and 40 keV energy levels, there was no statistically significant difference in CT (F = 0.401, *P* = 0.681/ F = 0.398, *P* = 0.683), SNR (F = 0.057, *P* = 0.945/ F = 0.174, *P* = 0.843), and CNR (F = 0.064, *P* = 0.938/ F = 0.163, *P* = 0.852) values among the three groups with different tube diameters. For the same tube diameter, the mean value of CT, SD, CNR and SNR at 40 keV were superior to those of conventional CT scanning (Table 5). Regardless of the energy level (conventional or 40 keV), the three tube diameters showed no significant difference in contrast (χ^2^ = 0.688, *P* = 0.709/ χ^2^ = 0.629, *P* = 0.730), acceptability (χ^2^ = 0.688, *P* = 0.709/ χ^2^ = 2.000, *P* = 0.368), or sharpness scores (χ^2^ = 0.000, *P* = 1.000/ χ^2^ = 0.000, *P* = 1.000).

**Table 5:** Objective evaluation indexes of different tube diameters at different energy levels

Value	2.5 mm		4 mm		5 mm	
	40 keV	Conventional	40 keV	Conventional	40 keV	Conventional
CT	626.21 ± 362.88	269.48 ± 156.72	816.48 ± 421.46	341.98 ± 176.48	829.14 ± 282.75	361.02 ± 118.61
SD	64.11 ± 27.46	33.65 ± 13.08	33.65 ± 16.20	16.68 ± 4.34	17.73 ± 8.59	12.33 ± 3.10
CNR	142.86 ± 124.60	47.68 ± 33.74	128.22 ± 88.89	43.60 ± 31.31	106.38 ± 37.51	40.75 ± 12.36
SNR	147.94 ± 126.02	47.38 ± 33.61	132.47 ± 90.90	44.00 ± 31.66	109.67 ± 37.14	40.81 ± 12.17

Values in the table are presented as mean ± standard deviation. Abbreviation: CT, computed tomography value. SD, standard deviation. SNR, signal-to-noise ratio. CNR, contrast-to-noise ratio

## Discussion

This study aimed to compare the lower extremity vascular image quality using low tube voltage (100 kVp), low CTDI_vol_ (2.5 mGy), low energy level (40 keV), and low iodine concentration (4 mgI/mL) with that of conventional tube voltage (120 kVp), CTDI_vol_ (5 mGy, 10 mGy), energy levels (70 keV, 100 keV), and iodine concentrations (8 mgI/mL, 12 mgI/mL, 16 mgI/mL) utilizing a second-generation spectral CT scan system. In addition, we compared the image quality of different vessel sizes (2.5 mm, 4 mm, and 5 mm) at low energy levels (40 keV) and conventional CT scans under low tube voltages and radiation doses. There were four findings: First, the results showed almost no difference between the subjective and objective scores for different tube voltages (100 kVp and 120 kVp). Under the same tube voltage conditions, there were no differences in subjective and objective scores (except for the CNR value) across different CTDI_vol_ levels. Second, subjective and objective scores at the low energy level (40 keV) were comparable to those at higher energy levels (70 keV) and conventional CT scans. Third, there were no differences in subjective score and objective score between the ultra-low iodine concentration (4mgI/mL) at low energy levels and the medium-high concentrations (12mgI/mL, 16mgI/mL) in conventional CT scans. Fourth, with 100 kVp, 2.5 mGy, and 40 keV, no differences in objective scores were observed among different vessel diameters (2.5, 4, and 5 mm), which was better than those of conventional CT scans.

This study showed minimal difference in subjective and objective scores between 100 kVp and 120 kVp tube voltages, indicating that low tube voltage scanning (100 kVp) can be used in DECT for lower-extremity vessel imaging without compromising image quality. Previously, spectral imaging was only possible at 120 kVp and 140 kVp [18], but the latest generation of detector-based DECT also allows acquisition at 100 kVp [14, 19]. Spectral imaging at 100 kVp not only increases intravascular CT attenuation but also effectively reduces radiation dose. This addresses issues related to poor vascular enhancement in patients with renal insufficiency, radiation exposure in pediatric CTA, and various other concerns [20, 21]. Yang et al. showed that DECT images at 100kVp and 50 keV enhanced the contrast of lower limb arteries and improved diagnostic accuracy for the inferior patellar artery. Their study reported a mean CTDI_vol_ of 5.7 mGy for DECT and 6.0 mGy for conventional CT, with a 12% reduction in radiation dose in DECT scans compared to conventional scans. Our study revealed no significant difference in image quality between the 2.5 and 5 mGy CTDI_vol_ groups. It should be noted that a radiation dose of 5 mGy is standard for lower-extremity clinical imaging [22]. A previous study comparing image quality metrics and dose exposure between low-dose and standard-dose lower-extremity CTA in 107 patients with PAD, reported mean CDTI_vol_ values of 4.1 ± 1.1 mGy for standard-dose CT and 2.9 ± 0.8 mGy for low-dose CT [22]. Although this study primarily evaluated quantitative image metrics and performance, our findings suggest that lower-dose exposure is feasible in a phantom setting while maintaining spectral imaging performance. These results need to be further investigated in clinical settings.

Our study also found that the subjective and objective scores of low-energy images (40 keV) were comparable to those at higher energy levels (70 keV) and conventional CT scans, and in some cases, they were even superior. A previous study demonstrated that CT images at energy levels below 60 keV could enhance the visualization of the lower-extremity arteries, improve image quality, and reduce contrast agent dose [23], which aligns with our findings. In this study, we evaluated the image quality of the lowest single-level image (40 keV) in displaying the ROI. We found that 40 keV images improved the CT value in the ROI and produced high-quality images with higher SNR and higher acceptability, contributing to improved imaging and diagnostic accuracy for CTA. At a low tube voltage (100 kVp) and low CTDI_vol_ (2.5 mGy), compared to the higher iodine concentrations (12 mgI/mL and 16 mgI/mL) used in conventional 100 kVp scans, the CT, SD, CNR, and SNR values at lower iodine concentrations (4 mgI/mL) at 40 keV showed no significant differences. This further confirms that 40 keV can improve CT values and significantly reduce contrast agent dosage, which is clinically meaningful for patients with renal failure who require CT examination. Moreover, reducing contrast agent dosages is economically beneficial for patients.

The visualization of lower extremity vasculature, particularly the distal microvessels, is of critical clinical importance. Known methods for improving small-vessel imaging include using low tube voltages [24], digital subtraction angiography (DSA), or magnetic resonance imaging (MRI) [25]. However, MRI has several contraindications, and its long examination times may cause patients discomfort and motion artifacts [26]. Additionally, as an invasive procedure, DSA is unsuitable for primary screening. The advantages of DECT include the ability to simultaneously generate anatomical and spectral images, reducing beam-hardening artifacts and image noise [19]. Our study’s favorable results indicate improved accuracy and precision in the display of small vessels with DECT, especially those with tube diameters as small as 2.5 mm.

Our results show that the image quality of 40 keV images displaying small vessels is superior to that of conventional 100kVp and 2.5mGy images. Previous studies have shown that 40 keV improves the ability to distinguish real blood vessel lumens from blood vessels [27], significantly improving the visualization of small blood vessels. The blood vessels of the lower extremities include ilio-abdominal, femoral, popliteal segments, and lower knee segments. The common femoral artery (CFA) is typically 4 cm long, with a diameter of 7.9 to 8.5 mm. The mean popliteal artery diameter is 5.9–6.2 mm, while infrapopliteal arteries, such as the posterior tibial artery, have a diameter of 2.0–2.2 mm [28, 29]. Our results demonstrate that 40 keV can significantly improve the image quality of vessels with a 2.5 mm tube diameter in a phantom study, which is clinically meaningful for bilateral lower-extremity CTA.

This study has some limitations. First, although the phantoms had heterogeneous inserts simulating real vessels, they were not anthropomorphic. Limited by the experimental conditions, this experiment lacks a longitudinal evaluation model to simulate blood vessels, and future research should be extended to more realistic models, such as 3D printed models designed specifically for DECT applications. Second, the minimum diameter achievable with this phantom was 2.5 mm, whereas distal lower limb vessels (e.g., the posterior tibial artery) can be as narrow as 2.0 mm. For lower-limb vessels, further studies using newer techniques to fabricate phantom with smaller vessel diameters should be conducted. Third, it is important to note that while our experimental results demonstrate the excellent image quality provided by 40 keV in a lower extremity vascular phantom, its clinical application might be limited by increased noise or artifacts in the context of obesity or vascular calcification. Furthermore, patient motion in clinical settings poses a persistent challenge to image quality. Phantom studies should consider motion artifact interventions, and subsequent studies could consider using a dynamic phantom to explore how to reduce these artifacts.

## Conclusion

In second-generation dual-energy spectral CT angiography of lower extremity vessels, the use of 40 keV energy level images at 100 kVp tube voltage can achieve image quality and diagnostic accuracy comparable to conventional CT angiography, with the additional benefit of lower contrast medium dosage and radiation dose (2.5 mGy), and enhances the visualization of small vessels.

## Supplementary Information

Below is the link to the electronic supplementary material.

Supplementary Material 1

## Data Availability

The datasets used and/or analysed during the current study are available from the corresponding author on reasonable request.

## Abbreviations

CFA: Common femoral artery
CM: Contrast medium
CNR: Contrast-to-noise ratio
CT: Computed tomography
CTA: Computed tomography angiography
CTDIvol: Computed tomography dose index volume
DECT: Dual-energy computed tomography
DSA: Digital subtraction angiography
MRI: Magnetic resonance imaging
PAD: Peripheral artery disease
ROI: Region of interest
SD: Standard deviation
SNR: Signal-to-noise ratio
VMI: Virtual monoenergetic images
