# Supplementary material for: Phantom-based quantification of the spectral accuracy of lower extremity vascular imaging using a second-generation dual-layer spectral CT at 100 kVp and 120kVp
Source: BMC Med Imaging. 2025 Dec 4;25:500. doi: 10.1186/s12880-025-02047-8 (PMC12676852; doi:10.1186/s12880-025-02047-8)
Supplement: Supplementary file 1 — Supplementary Material 1 [file 12880_2025_2047_MOESM1_ESM.docx]

**Table S1** Comparison of subjective evaluation indexes of different tube voltage at three different CTDI_vol_

| Item | 2.5 mGy | | 5 mGy | | 10 mGy | |
| --- | --- | --- | --- | --- | --- | --- |
|  | 100kVp (A)  (1/2/3/4/5) ^#^ | 120kVp (B)  (1/2/3/4/5) | 100kVp (C)  (1/2/3/4/5) | 120kVp (D)  (1/2/3/4/5) | 100kVp (E)  (1/2/3/4/5) | 120kVp (F)  (1/2/3/4/5) |
| Contrast | 3/3/10/17/15° | 2/7/9/13/17 | 2/3/9/19/15 | 1/2/9/20/16 | 3/3/9/17/16 | 3/3/10/14/18 |
| Sharpness | 0/0/28/20/0 | 0/0/25/23/0 | 0/3/28/17/0 | 0/0/32/16/0 | 0/0/33/15/0 | 0/0/27/21/0 |
| Subjective noise | 0/0/4/44/0 | 0/0/0/48/0 | 0/0/0/48/0 | 0/0/0/48/0 | 0/0/0/48/0 | 0/0/0/48/0 |
| Acceptability | 3/4/8/31/2 | 2/7/9/28/2 | 2/3/9/33/1 | 1/4/9/34/0 | 3/3/9/31/2 | 3/4/8/32/1 |

^#^, (1/2/3/4/5) represents the scores of each subjective evaluation index. °, For example, 3/3/10/17/15 represents the number of each subjective score.

**Table S2** Comparison of objective evaluation indexes of different tube voltage at three different CTDI_vol_

| Value | 2.5 mGy | | | 5 mGy | | | 10 mGy | | |
| --- | --- | --- | --- | --- | --- | --- | --- | --- | --- |
|  | 100 kVp(A) | 120 kVp(B) | P value | 100 kVp(C) | 120 kVp(D) | P value | 100 kVp(E) | 120 kVp(F) | P value |
| CT | 351.32±312.23 | 329.91±323.48 | 0.742 | 346.72±336.76 | 335.15±329.25 | 0.865 | 351.53±336.75 | 336.17±332.70 | 0.823 |
| SD | 20.99±18.23 | 20.57±24.33 | 0.926 | 20.82±21.82 | 19.77±24.75 | 0.826 | 20.79±21.87 | 19.36±28.61 | 0.784 |
| CNR | 58.95±59.93 | 59.96±65.93 | 0.937 | 74.72±73.99 | 77.40±76.43 | 0.862 | 90.67±88.00 | 103.08±107.13 | 0.537 |
| SNR | 59.01±62.05 | 60.22±67.45 | 0.928 | 74.56±76.52 | 77.68±78.38 | 0.844 | 90.47±90.31 | 103.55±109.36 | 0.524 |

Abbreviation: CT, computed tomography value. SD, standard deviation. SNR, signal-to-noise ratio. CNR, contrast-to-noise ratio.

**Table S3** Comparison of objective evaluation indexes of different tube voltage at three different CTDI_vol_

| Value | 100 kVp | | |  | 120 kVp | | |  | |  | |
| --- | --- | --- | --- | --- | --- | --- | --- | --- | --- | --- | --- |
|  | 2.5mGy(A) | 5mGy (C) | 10mGy(E) | P value | 2.5mGy(B) | 5mGy(D) | 10mGy(F) | | P value | |  |
| CT | 351.32±312.23 | 346.72±336.76 | 351.53±336.75 | 0.997 | 329.91±323.48 | 335.15±329.25 | 336.17±332.70 | | 0.995 | |  |
| SD | 20.99±18.23 | 20.82±21.82 | 20.79±21.87 | 0.999 | 20.57±24.33 | 19.77±24.75 | 19.36±28.61 | | 0.973 | |  |
| CNR | 58.95±59.93 | 74.72±73.99 | 90.67±88.00 | 0.120 | 59.96±65.93 | 77.40±76.43 | 103.08±107.13^#^ | | 0.047* | |  |
| SNR | 59.01±62.05 | 74.56±76.52 | 90.47±90.31 | 0.140 | 60.22±67.45 | 77.68±78.38 | 103.55±109.36 | | 0.052 | |  |

*At 120kV tube voltage, the CNR values of the three different CTDI_vol_ groups (group A, B and C) were statistically different (P=0.047). ^#^Pairwise comparisons with Bonferroni correction showed significant differences from group B. Abbreviation: CT, computed tomography value. SD, standard deviation. SNR, signal-to-noise ratio. CNR, contrast-to-noise ratio.
